# A Pilot Analysis of Circulating cfRNA Transcripts for the Detection of Lung Cancer

**DOI:** 10.3390/diagnostics12122897

**Published:** 2022-11-22

**Authors:** Chamindi Seneviratne, Amol Carl Shetty, Xinyan Geng, Carrie McCracken, Jessica Cornell, Kristin Mullins, Feng Jiang, Sanford Stass

**Affiliations:** 1 Department of Psychiatry, University of Maryland School of Medicine, Baltimore, MD 21201, USA; 2 The Institute for Genome Sciences, University of Maryland School of Medicine, Baltimore, MD 21201, USA; 3 Department of Pathology, University of Maryland School of Medicine, Baltimore, MD 21201, USA; 4 Laboratories of Pathology, University of Maryland Medical Center, Baltimore, MD 21201, USA

**Keywords:** NSCLC, cell-free RNA, liquid biopsies, biomarkers, smokers, plasma, circulating RNA

## Abstract

Lung cancers are the leading cause of cancer-related deaths worldwide. Studies have shown that non-small cell lung cancer (NSCLC), which constitutes the majority of lung cancers, is significantly more responsive to early-stage interventions. However, the early stages are often asymptomatic, and current diagnostic methods are limited in their precision and safety. The cell-free RNAs (cfRNAs) circulating in plasma (liquid biopsies) offer a non-invasive detection of spatial and temporal changes occurring in primary tumors since the early stages. To address gaps in the current cfRNA knowledge base, we conducted a pilot study for the comprehensive analysis of transcriptome-wide changes in plasma cfRNA in NSCLC patients. Total cfRNA was extracted from archived plasma collected from NSCLC patients (N = 12), cancer-free former smokers (N = 12), and non-smoking healthy volunteers (N = 12). Plasma cfRNA expression levels were quantified by using a tagmentation-based library preparation and sequencing. The comparisons of cfRNA expression levels between patients and the two control groups revealed a total of 2357 differentially expressed cfRNAs enriched in 123 pathways. Of these, 251 transcripts were previously reported in primary NSCLCs. A small subset of genes (N = 5) was validated in an independent sample (N = 50) using qRT-PCR. Our study provides a framework for developing blood-based assays for the early detection of NSCLC and warrants further validation.

## 1. Introduction

Lung cancers are the leading cause of cancer-related deaths in both men and women in the U.S. and worldwide. Non-small cell lung cancer (NSCLC) constitutes approximately 84% of all lung cancer cases and consists of two main histological subtypes: adenocarcinoma (AC) and squamous cell carcinoma (SCC) [1]. The main risk factor for developing NSCLC is smoking, which is preventable yet highly prevalent with over a billion smokers around the world [2]. Moreover, smoking and other environmental pollutants interact with biological factors such as aging and genetic risk variants to increase disease burden [3,4,5,6]. Furthermore, the NSCLC risk has been shown to correlate positively with the severity and duration of smoking and negatively with the time since smoking cessation [7,8].

Because lung cancers are often asymptomatic in early stages, most patients are diagnosed at advanced stages, resulting in only about 15–20% of patients surviving five years after diagnosis [6]. Early-stage NSCLCs are more responsive to treatment [9] and are, therefore crucial to reducing mortality. At present, the only recommended diagnostic method for NSCLC is the detection of pulmonary nodules (PNs) with low-dose computed tomography (LDCT) [10]. In fact, based on data from the Cancer Intervention and Surveillance Modeling Network (CISNET), the US Preventive Services Task Force (USPSTF) recommended the annual screening of adults aged from 50 to 80 years of age with a smoking history of 20 or more pack-years and who currently smoke or quit smoking within the past 15 years [11]. This 2021 USPSTF recommendation (A-50-80-20-15) was updated to expand the population eligible for LDCT screening over the previous 2013 USPSTF recommendation that required a smoking history of 30 or more pack-years (A-50-80-30-15). The LDCT has high negative predictive values, moderate sensitivity and specificity, and low positive predictive values [12]. A recent meta-analysis corresponding to data from 84,558 participants who had a smoking history of 15 or more pack-years indicated a 17% relative reduction in mortality in the group screened with LDCT compared with the control group [12]. Despite these encouraging statistics, there are several important limitations to using LDCT for NSCLC diagnosis. For example, the high false-positive rates can lead to the further testing of benign PNs with invasive diagnostic and therapeutic procedures such as serial CTs, biopsy, and surgery that carry their own morbidities. These invasive procedures are reported to be performed in 44% of smokers with indeterminate PNs that have, roughly, a 5% probability of malignancy, and 35% of surgical resections are ultimately determined to be benign diseases [13]. Another concern is the exposure to radiation with repeated LDCT. Statistical modeling has predicted 1 death for every 13.0 lung-cancer-related deaths avoided by LDCT with 2021 USPSTF recommendations, which was a 2% worsening compared to the risk associated with 2013 USPSTF recommendations [11]. Considering these factors, it is clinically important to develop noninvasive biomarkers to distinguish malignant from benign PNs, facilitating positive screening results when using LDCT.

Recently, the concept of liquid biopsies has garnered excitement among the scientific community for its potential to provide real-time information on spatial and temporal changes in tumor markers in an easily obtained peripheral blood sample [14]. Several types of biomarkers have been explored in liquid biopsies as potential diagnostics with mixed results. Circulating tumor DNAs (ctDNAs) have over 90% sensitivity and specificity for NSCLC diagnosis in patients with stage II–IV NSCLC but around 50% in patients with stage I NSCLC when shedding rates are low [15]. The analysis of mutations in ctDNA has also been reported to have a lower sensitivity and specificity in early-stage NSCLC [16]. Therefore, analyses of ctDNA mutations or quantities appear to be more suitable for therapeutic and disease monitoring in NSCLC patients rather than early detection. In contrast, tumors with low shedding rates add cell-free RNAs (cfRNAs) to blood circulation, presenting us with the opportunity to identify the overexpressed, tumor-specific, and tumor-derived RNA signals in the blood [17] at early stages, potentially facilitating high rates of patients that are able to receive curative surgical resections. Studies have also shown that cfRNA could complement ctDNA and thus improve early diagnosis [18]. The studies of cfRNA have mainly focused on either microRNAs (miRNAs) or a small number of known cancer-related messenger RNAs (mRNAs) [19,20,21]. Moreover, the published studies used large amounts of plasma—up to 4–5 mL—for cfRNA extraction for expression analyses, limiting its potential clinical use. We have conducted a pilot study to explore the ability to detect cfRNA signatures of NSCLC, particularly of the genes that were previously reported to be differentially expressed in lung cancer primary tissue biopsies, compared with both cancer-free smokers and healthy non-smokers.

## 2. Materials and Methods

Study design: In this pilot study, we first compared the expression levels of plasma cfRNA obtained from SCC and AC patients (N = 12; cases) and cancer-free former smokers (N = 12; control_smokers). As all patients in the case group were also heavy smokers, we included a second control group of non-smoking healthy individuals (N = 12; control_healthy) to exclude differentially expressed cfRNAs associated with smoking, rather than pathological processes underlying NSCLC. Each participant provided whole blood samples as part of an umbrella protocol approved by the Institutional Review Board of the University of Maryland Baltimore [UMB IRB protocol ID: HP-00040666] and the Veterans Affairs Maryland Health Care System. All participants provided written informed consent to participate in the research conducted at the University of Maryland Medical Center and the Baltimore VA Medical Center. Diagnosis of lung cancer was established by the pathological examination of tissues obtained via surgery or biopsy. Histological diagnoses were made on bronchoscopic biopsy specimens and thoracotomy according to the World Health Organization (WHO) categories. The NSCLC stage classification was based on the WHO classification and the International Association for the Study of Lung Cancer staging system. The smokers consisted of former smokers who had a minimum smoking history of 30-pack years and quit within the past 15 years. The exclusion criteria were similar to Leng et al. 2017 [8]. The demographic and clinical characteristics of the cohorts are presented in Table 1.

Sample preparation and sequencing: The archived plasma samples (volumes given in Table 1) prepared from 3–6 mL of whole blood collected into tubes containing EDTA were thawed at 37 °C and centrifuged at 16,000× *g* for 30 min at 4 °C to remove any cellular components in the plasma. The supernatant was extracted and centrifuged again at 13,000× *g* for 30 min at 4 °C and stored at −80 °C until the day of cfRNA extractions. The quality control procedures for the plasma sample preparations were similar to our earlier study [22]. cfRNA was extracted from archived plasma samples using the miRNeasy^®^ Serum/Plasma Advanced Kit (Qiagen) according to the manufacturer’s guidelines and was tested for RNA integrity using an *Agilent* bioanalyzer system. The libraries were prepared using a tagmentation-based method consisting of a two-step probe-assisted exome enrichment for cfRNA detection (Illumina, Inc, San Diego, CA) [23]. An Illumina Exome enrichment panel that included >425,000 probes (oligos), each constructed against the NCBI37/hg19 reference genome, covering >98% of the RefSeq exome was used to pool libraries with the target cfRNAs of interest. The probe set was designed to capture >214,000 targets, spanning 21,415 genes of interest. The probes hybridized to target the libraries were captured according to protocol and amplified using a 19-cycle PCR program. The enriched libraries were then purified with magnetic beads and then sequenced using a NovaSeq 6000 system (Illumina, Inc) at a sequencing depth of 100 million reads at 100 bp PE length sequences.

Sequencing data analyses: The raw sequence reads generated for each sample were analyzed using the CAVERN analysis pipeline [24]. Read quality was assessed using the FastQC toolkit to ensure good-quality reads for downstream analyses. The reads were aligned with the human reference genome GRCh38 (available from the *Ensembl* repository) using HISAT2, a fast splice-aware aligner for mapping next-generation sequencing reads [25]. The reads were aligned using default parameters to generate the alignment BAM files. The read alignments were assessed to compute gene expression counts for each gene using the HTSeq count tool [26] and the human reference annotation (GRCh38). The raw read counts were normalized for library size and dispersion of gene expression. The normalized counts were utilized to assess the differential cfRNA expression between conditions using DESeq2. The *p*-values were generated using the Wald test implemented in DESeq2 and then corrected for multiple hypothesis testing using the Benjamini–Hochberg correction method [27]. The significant differentially expressed cfRNAs between conditions were determined using a false discovery rate (FDR) of 5% and a minimum absolute log2 (fold-change) of 1.

Quantitative RT-PCR (qRT-PCR) for validation of a subset of cfRNA: Based on the findings from the sequencing data analyses, we selected five differentially expressed protein-coding genes, as listed in Table 1 and detailed below in the results section for validation assays. We assessed the abundance of cfRNA for the five selected genes using qRT-PCR in an independent set of plasma samples from 25 cases (AC = 13; SCC = 12) and 25 controls (control_smokers = 18; control_healthy = 7). The demographic and clinical characteristics of the validation cohort are presented in Table 1. Total cfRNA was extracted from archived plasma samples (500 uL per sample) using the same protocol described above for the discovery cohort. A mixture of three commercially available RNA spike-ins (miRNAs UniSp2, UniSp4, and UniSp5) from the *RNA Spike-In Kit, For RT* was added to the plasma samples according to the manufacturer’s protocol (Qiagen, Germantown, MD, USA) prior to the extraction of cfRNA to control for cfRNA isolation across the samples. The extracted total cfRNA samples were then split into equal volumes for cDNA synthesis and the subsequent mRNA quantification and detection of the three miRNA spike-ins in parallel. We used *miRCURY LNA RT* and *miRCURY LNA SYBR Green PCR* kits (Qiagen) for the reverse transcription and qPCR of spike-in miRNAs and the *QuantiTect^®^ Reverse Transcription* and *QuantiTect SYBR Green RT-PCR* kits (Qiagen) for the reverse transcription and qPCR of the selected protein-coding genes. All qPCR reactions were performed in triplicates with 1:10 cDNA dilutions in a Bio-Rad CFX real-time PCR detection system (Bio-Rad, Hercules, California, USA), according to the protocols associated with each kit. As stable endogenous reference genes for quantifying circulating mRNA in plasma samples have not been established in the literature and normalizing to a global mean of all expressed mRNA was not applicable to the analyses of five genes, we opted not to use a reference gene in this pilot study. We also explored the possibility of using GAPDH—the commonly used endogenous reference gene for cellular mRNA—and did not detect any amplification. Therefore, we adopted a method of, first, assessing the between-sample variability using three spike-ins to identify outlier samples and then performing qRT-PCR for the five selected genes, excluding outliers. Two-tailed t-tests using GraphPad Prism software (San Diego, CA, USA) were performed for statistical comparisons.

## 3. Results

cfRNA processing and quality control: cfRNA was extracted from all 36 samples at mean concentrations of 0.111 ng/uL in cases, 0.085 ng/uL in control_smokers, and 0.151 ng/uL in control_healthy. The RNA integrity numbers (RIN) ranged from 1 to 5.3. All samples had sequence reads that mapped >80% to the reference sequence and mapped to the exonic regions. Total Gene Abundance ranged from approximately 10 to 70 million. Of these genes, 0.5–10% were Hb coding genes, 0.5–20% mitochondrial genes, <0.03% ribosomal RNA (rRNA) genes, and up to 4% were other non-coding RNA (ncRNA) genes. Amongst the protein-coding genes, the most abundant were actin, myosin, platelet-specific genes, and pseudogenes.

**Figure 1:**
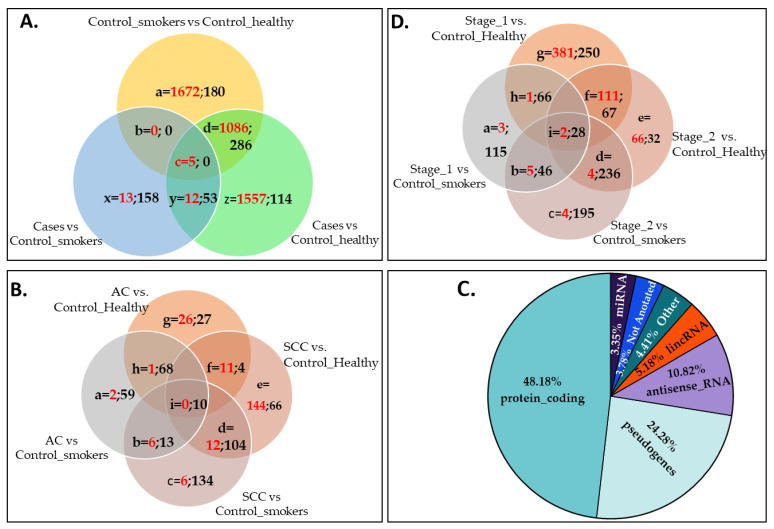
Distribution of NSCLC-associated cfRNA. (**A**): cfRNA in plasma samples from cases. (**B**): cfRNA in subtypes AC and SCC. (**C**): Distribution of NSCLC-associated cfRNA within functional categories. The most common pseudogene subcategories were processed_pseudogenes (17.99%), unprocessed_pseudogenes (2.89%), and transcribed_unprocessed_pseudogenes (1.82%), and other subtypes were present <1%. The “Other” category included the following subcategories at less than 1% abundance: IG_V_genes, snoRNA, processed_transcripts, TR_V_genes, TR_J_genes, sense_intronic, misc_RNA, scaRNA, sense_overlapping, IG_C_genes, TR_C_genes, 3prime_overlapping_ncRNA, IG_J_genes, TEC, and TR_D_genes. (**D**): cfRNA within categories based on NSCLC stage. The numbers presented in red and black color fonts in Figure 1A–C represent up- and down-regulated genes, respectively.

**Figure 2:**
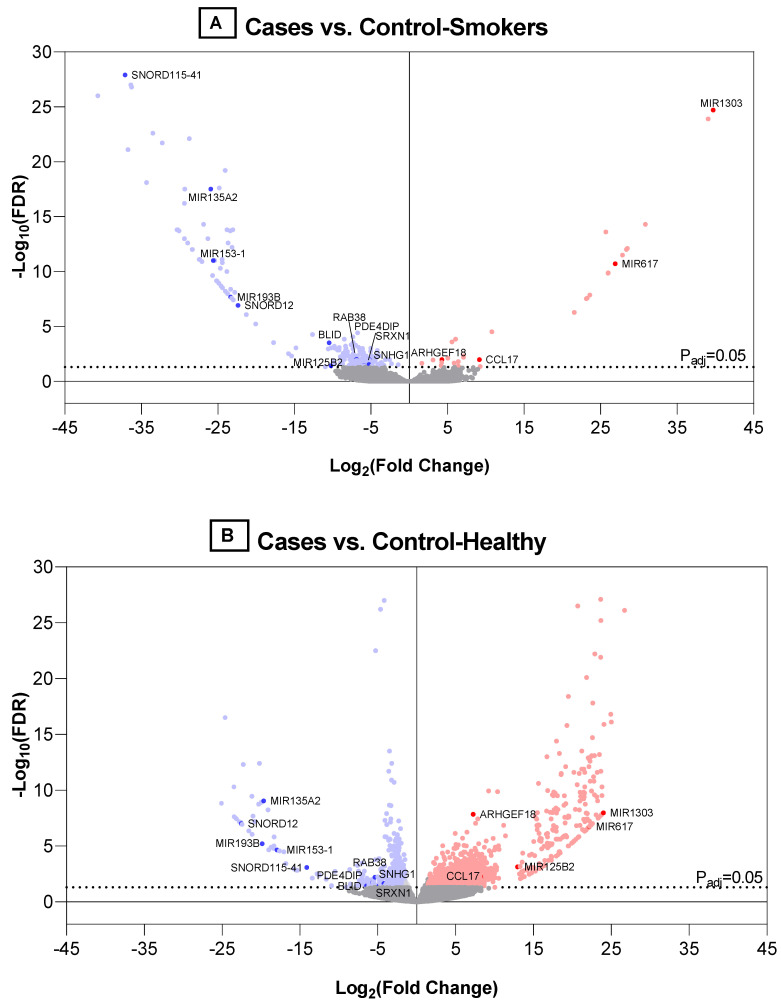
Volcano plots for (1) cases vs. smokers with benign PN (**A**) and (2) cases vs. healthy non-smokers (**B**). The horizontal dotted lines indicate an adjusted *p*-value of 0.05. The dots are colored blue or red if classified as down- or up-regulated, respectively, using a threshold of log 2-fold change of −1 and 1.

Identification of differentially expressed cfRNAs between cases and controls: The differential expression of cfRNA was analyzed after excluding Hb, mitochondrial, and rRNA transcripts. As shown in Figure 1A, a total of 1905 (x + y + z) cfRNAs were identified to be differentially expressed in the plasma samples from cases compared with the two control groups. Of these, two cfRNAs (LINC01956 and TAS2R16) were differentially expressed in opposite directions in cases compared with the control_smokers and control_healthy groups, and, therefore, we have included these in both the x and z categories in Figure 1A. Both cfRNAs were downregulated compared with the control_smokers group and upregulated compared with the control_healthy group. Another 1377 (b+c+d in Figure 1A) cfRNAs that were detected in cases were differentially expressed in the same direction in cancer-free smokers. The volcano plots for the comparison of cfRNA differential expression between cases and controls are presented in Figure 2A,B.

Statistical power analysis: The post hoc power analysis revealed that the samples of 12 cases and 24 controls afforded a 78.5% power to detect differentially expressed genes with a 2-fold effect size using a 5% false discovery rate.

Exploratory subgroup analyses: We performed two subgroup analyses exploring the differentially expressed cfRNAs between (1) subtypes of cases, AC vs. SCC, and (2) based on NSCLC stages, stages I vs. II, compared with both control groups, irrespective of their statistical significance in the combined case group. Figure 1B presents all cfRNAs within each subtype category excluding DEGs shared with cancer-free smokers (i.e., comparisons between the control_smokers and control_healthy groups). Of these, a total of 452 cfRNAs (64.3% of all DEGs in Figure 1B) were not detected in the combined cases (x + y + z in Figure 1A) but uniquely differentially expressed in either AC or SCC, or both, but in differing directions. As depicted in Figure 1C, nearly half of all 2357 total cfRNAs (1905 + 452) were functional protein-coding genes (Figure 1C). All the cfRNAs included in Figure 1 are listed in Supplementary Table S1. Similarly, Figure 1D presents cfRNA comparisons between NSCLC stages I and II, excluding cfRNAs shared with cancer-free smokers. Comparisons with other NSCLC stages were not possible as we had only one sample from a patient diagnosed with stage III and none for stage IV. The results indicated that 1075 genes were expressed in plasma from patients who had stage I NSCLC (a+b+h+i+g+f in Figure 1D), out of which 259 were common to both stages I and II. As both subgroup analyses had small numbers of patients within each category (Table 1), these findings should only be considered as exploratory.

**Figure 3:**
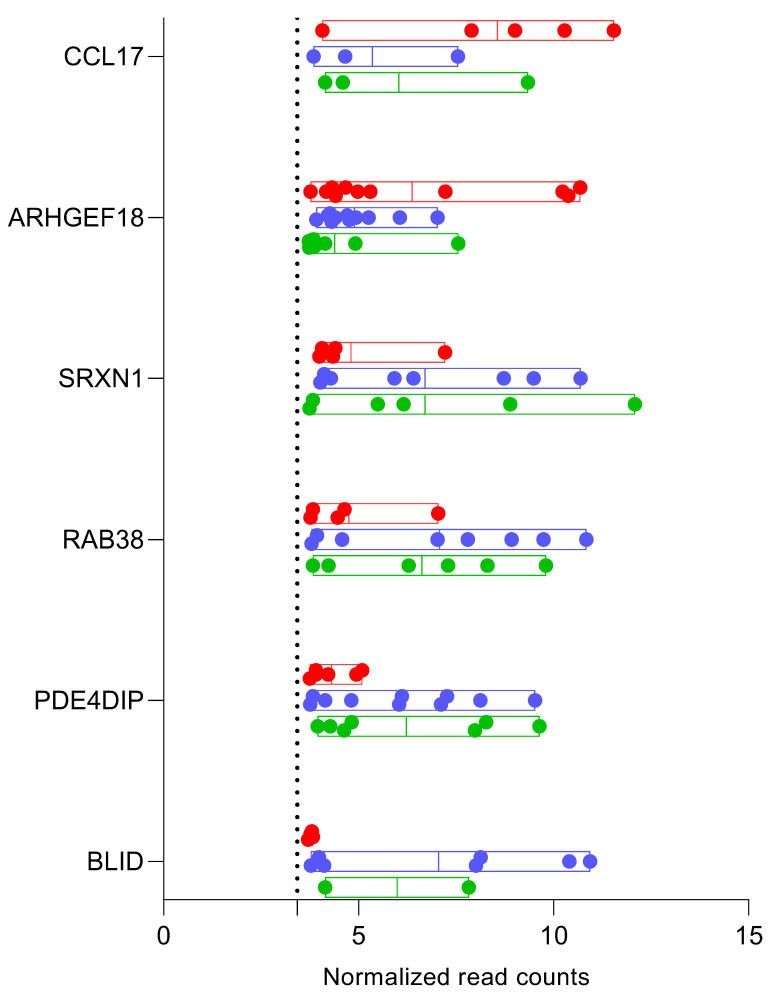
Distribution of read counts across individual samples for cfRNA of replicated protein-coding genes. Each dot represents cfRNA read counts for a given gene within individual samples. Red—genes in cases; blue—smokers with benign PN; green—healthy non-smokers. The dotted line represents the threshold for detecting read counts that was set at 3.4298.

Literature review to identify DEGs previously reported in primary NSCLC biopsies: We performed an exhaustive review of all the published studies listed on the National Center for Biotechnology Information (NCBI)’s database for gene-specific information, using gene IDs for each of the 2357 identified DEGs. Studies reporting DEGs in primary NSCLC biopsies were identified and are referenced in Supplementary Table S1. Our literature review showed that 10.65% of the total DEGs (N = 251 of 2357) have been reported in primary tumor biopsies from NSCLC patients in the published studies. The majority of these replicated genes were mRNA transcripts of protein-coding genes (N = 174; 69.32%), while some (N = 45; 17.92%) were miRNA. Next, to assess the inter-patient variation in cfRNA transcript abundance within each group (i.e., combined cases, control_smokers, and control_healthy), we evaluated whether the transcripts were expressed above detectable levels and then calculated the coefficient of variation (%CV) within a group for each gene. Of the total 174 replicated protein-coding genes identified in this study, 78.97% were expressed above the threshold in cases and 88% had <50% CV for each replicated gene (Supplementary Table S1). Fifteen cfRNAs that were differentially expressed in cases compared with both control groups (category “Y” in Figure 1A) and reported in primary NSCLC tissue biopsies are listed in Table 2. The distribution of these 15 replicated cfRNAs that were differentially expressed in cases compared with the two control groups are marked in volcano plots presented in Figure 2A,B. Of the six replicated protein-coding genes, all but CCL17 were expressed with <50% CV in the samples within cases (Table 2 and Figure 3). Therefore, we selected the five genes (i.e., ARHGEF18, SRXN1, RAB38, PDE4DIP, and BLID) for further validation in an independent cohort.

**Figure 4:**
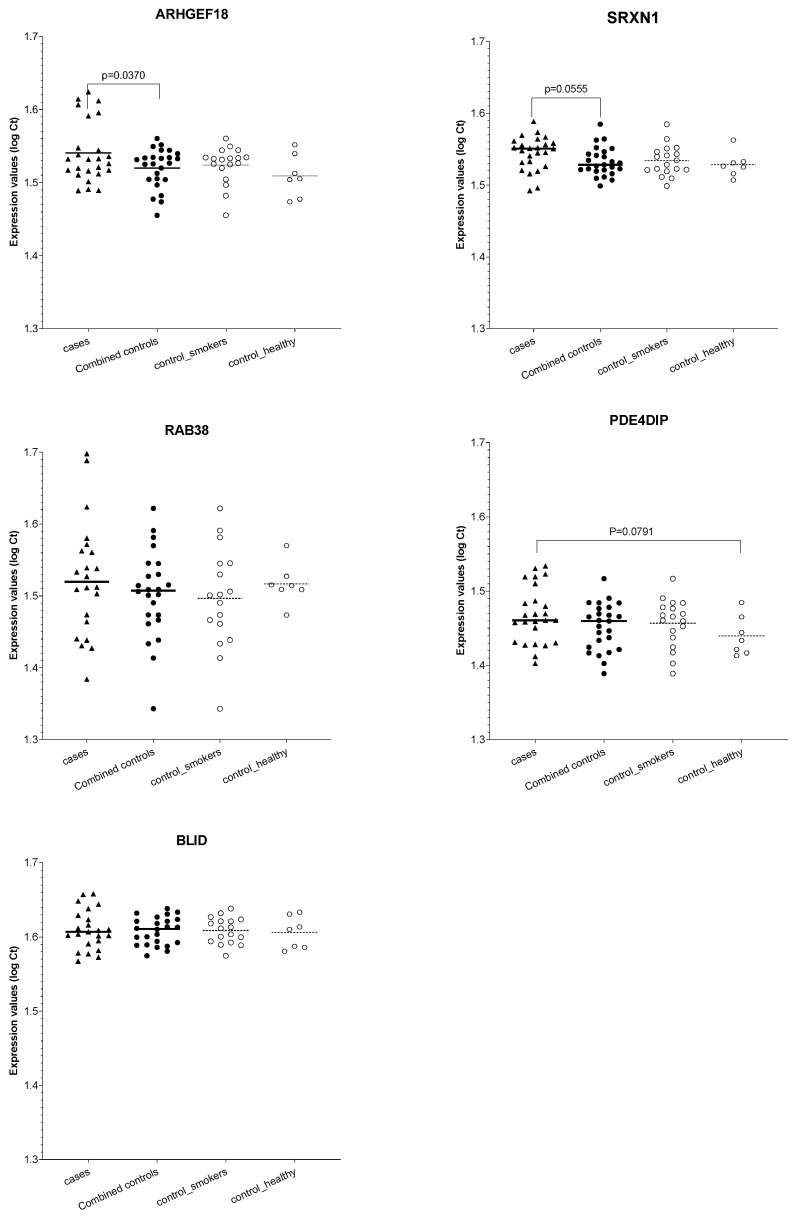
qRT-PCR analysis of changes in the expression levels of cfRNA of selective protein-coding genes in an independent sample. Each symbol represents log transformed Ct values of mRNA levels within each sample averaged across three technical repeats. The horizontal lines represent mean expression levels within each group.

Quantitative RT-PCR (qRT-PCR) for validation of replicated cfRNA of protein-coding genes: While all the listed genes in Table 2 are reported to underly the pathophysiology of NSCLC, we specifically selected the protein-coding genes for our initial validation, as the circulating mRNA was the most abundant type of cfRNA present in our discovery cohort, and cfmRNAs are relatively less characterized in the literature despite their biological relevance. The expression data for the three spike-ins in all 50 samples are presented in Supplementary Figure S1. As UniSp2, UniSp4, and UniSp5 were detected in all samples, we assessed the cfmRNA for the five genes in all 50 samples without excluding any. As shown in Figure 4, our findings indicated that three of the five tested genes were differentially expressed between cases and the controls. ARHGEF18 showed a nominally significant downregulation (i.e., higher Ct values) in cases (*p* = 0.037), and SRXN1 showed a trend towards downregulation in cases (*p* = 0.056) compared with the combined control group. PDE4DIP showed a trend towards downregulation in cases compared only with the healthy non-smokers (*p* = 0.079). The other two genes, RAB38 and BLID, did not show statistically significant expressed cfRNA levels between cases and the controls.

Gene ontology (GO) enrichment analysis of differentially expressed cfRNA: The unbiased pathway analysis with cfRNA for the differentially expressed genes included in each category of Figure 1A revealed 123 significantly enriched pathways across the three comparison groups. Cases compared with the control_smokers group had one significantly enriched pathway that was also detected in cancer-free smokers; GO:0010629 (negative regulation of gene expression) with 286 cfRNAs in the control_smokers vs. control_healthy groups (adjusted *p* = 0.0041) and 24 cfRNAs in cases vs. control_smokers group (adjusted *p* = 5.98 × 10^−5^). However, at an individual gene level, only two cfRNAs (MIR874 and MIR551B) in GO:0010629 were common to the two groups, both in terms of direction and type. The cases vs. control_smokers and cases vs. control_healthy comparisons did not share any significantly enriched pathways. Eighty-five pathways were commonly enriched in cases and cancer-free smokers when each group was compared with the control_healthy group. Details of the 37 pathways that were uniquely enriched in cases compared with both control groups include general mechanisms underlying cancer biology and are presented in Table 3 below. The gene IDs for the cfRNAs enriched within these pathways are listed in Supplementary Table S2.

Twenty-five of thirty-seven uniquely enriched pathways in cases were compared against the non-smoking control group, of which twenty were in the GO domain of biological process (BP) and five in the domain of molecular function (MF). For the BP domain the significant terms were: GO:0001501, GO:0007186, GO:0007200, GO:0007399, GO:0008154, GO:0009888, GO:0009953, GO:0010454, GO:0032501, GO:0042221, GO:0042246, GO:0042692, GO:0043403, GO:0043503, GO:0045165, GO:0051272, GO:0051493, GO:1902903, GO:1904888, and GO:2001046. For the MF domain, the significant terms were: GO:0005125, GO:0005198, GO:0019958, GO:0030545, and GO:0048018. The remaining 12 of the 37 pathways uniquely enriched in cases were compared against the cancer-free smokers. These were in the BP (N = 7), MF (N = 2), and cellular component (CC; N = 3) domains. For the BP domain, the significant terms were: GO:0010608, GO:0016441, GO:0016458, GO:0031047, GO:0035194, GO:0035195, and GO:0040029. For the MF domain, the significant terms were: GO:0003729 and GO:1903231. For the CC domain, the significant terms were: GO:0016442, GO:0031332, and GO:1990904.

## 4. Discussion

Various subtypes of circulating cfRNA have been tested in plasma for the early-stage detection of NSCLC. Building upon these studies, we performed a comprehensive analysis of circulating plasma cfRNA using next-generation sequencing technologies to expand the repertoire of non-invasively measurable NSCLC signatures. We identified 2357 cfRNAs enriched in 123 pathways in those with a diagnosis of NSCLC compared with the control groups consisting of cancer-free smokers and non-smokers. Nearly half of the detected cfRNAs were transcripts of protein-coding genes, and 251 of the 2357 cfRNAs (10.65%) conformed to previously reported differentially expressed genes found in primary tumor biopsies from NSCLC patients. A majority (174 of 251) of these replicated transcripts were protein-coding genes, while the rest were previously reported miRNAs and other non-coding RNAs. In fact, two of the snoRNAs—SNORD115-41 and SNORD12—were previously reported in NSCLC tissue biopsies by our group [22].

Importantly, our pilot study used a workflow that can be easily adopted to develop a clinical assay for profiling cfRNA using plasma volumes smaller than those that have been reported elsewhere [56]. The archived plasma samples were derived from whole blood collected in standard 3–6 mL EDTA collection tubes routinely used in clinical care. The processing of small amounts of plasma (approximately 1.5 mL) yielded less than 5 ng of total cfRNA, and the library preparation with enrichment and sequencing was carried out for the efficient identification of cfRNA. Our methodology produced from 200 to 350 millions of sequence reads per sample, with over 80% of the reads mapping onto the exonic regions of the reference, comparable to what was reported with methods that required much higher volumes of plasma [57]. 

Although identifying biomarker signatures associated with NSCLC was not the primary objective of this proof-of-concept pilot study that sought to test the potential of an NGS-based method for the comprehensive detection of circulating cfRNA in plasma, we further evaluated the cfRNA of the 251 genes to explore potential candidates for future NSCLC-associated biomarker development studies. We first searched for cfRNAs that were differentially expressed in the plasma samples from NSCLC patients (regardless of the subtypes) compared with both smokers with benign PNs and non-smokers. Our results indicated fifteen genes that included six protein-coding, six miRNA, and three other non-coding genes. Twelve of the fifteen genes had low inter-patient variabilities (i.e., CV <50%) for cfRNA expression. These included five cf-mRNAs (ARHGEF18, RAB38, PDE4DIP, BLID, and SRXN1), four cf-miRNAs (MIR135A2, MIR193B, MIR617, and MIR125B2), and all three of the other non-coding genes (SNORD115-41, SNORD12, and SNHG1). Notably, the cfRNA for the two snoRNAs, genes SNORD115-41 and SNORD12, which we have previously reported [22], were not detectable in any NSCLC sample but were present in both control groups with low inter-subject variabilities, confirming their potential role as plasma biomarkers of NSCLC. Furthermore, identifying protein-coding genes (i.e., cf-mRNA) with low inter-patient variabilities was particularly significant as studies on circulating cf-mRNA are relatively sparse compared to miRNA or other non-coding genes. Thus, we tested the differential expression of the five cf-mRNAs associated with NSCLC in a different cohort of NSCLC patients, smokers with benign PN, and non-smokers using quantitative RT-PCR. Our results indicated a differential expression of cfRNA for the ARHGEF18, PDE4DIP, and SRXN1 genes but not RAB38 and BLID. The ARHGEF18 (Rho/Rac Guanine Nucleotide Exchange Factor 18), also known as P114-RhoGEF, activates the downstream gene RhoA, which is important for cell migration and tumor progression [58,59]. Song et al. showed that the ARHGEF18 gene was upregulated in squamous-cell carcinoma compared to adenocarcinoma or nontumor tissue and was significantly associated with lung cancer lymph node metastasis [31]. In line with these findings, we detected an upregulation of ARHGEF18 in our discovery cohort (Figure 3 and Table 2) but a downregulation in the validation sample (Figure 4). It is possible that the reversal in the direction of expression levels in the validation cohort occurred due to suboptimal qRT-PCR assay conditions as described below, rather than due to biological differences. The PDE4DIP (Phosphodiesterase 4D Interacting Protein) that anchors phosphodiesterase in centrosomes [35] was shown to co-express with the endogenous tumor suppressor gene THBS1, and high expression levels of PDE4DIP were associated with improved survival rates in adenocarcinoma patients [34]. Additionally, an exome-wide study of peripheral blood samples identified a frame-shift mutation in the PDE4DIP of cancer patients but not in cancer-free family members, suggesting a possible association of PDE4DIP with the development of squamous cell lung cancer [35]. The SRXN1 (Sulfiredoxin 1), another phosphodiesterase 4D anchoring protein, was found to be upregulated in the lung cancer cell lines A549 and 95D and 75 NSCLC tissues compared with the adjacent non-tumor tissue. In our study, both PDE4DIP and SRXN1 were downregulated in the discovery and validation cohorts [39]. More studies are needed to characterize the directionality associated with the clinical characteristics of NSCLC development and progression.

Our pilot study has several limitations. First, biological factors such as gender and age have been shown to play a major role in the development and prognosis of lung cancers [60]. For example, women smokers have a greater risk for developing lung cancer compared to men who smoke, presumably due to underlying genetic and other biological differences between men and women [61,62]; the AC subtype predominates in women, whereas SCC is more common in men [63]; and individuals aged 65 and older are at greater risk of developing lung cancers [60]. The over-representation of samples from male patients, when compared with the two control groups, and the modest sample size in this pilot project limited our ability to explore the moderating effects of these biological factors on our findings. This is particularly true of the subtype analyses that revealed 452 differentially expressed cfRNAs between the AC and SCC groups and 1075 between stages I and II that consisted of small numbers of patients. Second, both groups of smokers—with and without cancer—were significantly older than the non-smoking control group in the discovery cohort. The larger numbers of DEGs that we detected in comparisons of NSCLC patients and non-cancer smokers with non-smokers may, possibly, have arisen due to the confounding effects of age-related alterations in the expression of genes (see Figure 1A). However, we were able to validate three out of five selected genes tested in an independent cohort with a balanced age distribution between comparison groups. Third, because of a lack of information on stable endogenous reference gene(s) for the normalization of qRT-PCR data for circulating mRNA, we conducted validation analyses for the subset of five genes without the use of an endogenous control. Systematic analyses are urgently required to identify candidate genes with stable expression levels of cf-mRNA across samples for continued research on cf-mRNA analysis in NSCLC. Perhaps large RNA-seq data sets on circulating transcriptomes in plasma from NSCLC patients could facilitate such analyses. Fourth, we were not able to test the tissue specificity of the identified cfRNA because of the unavailability of lung tissue biopsies from the included participants for direct comparisons with plasma cfRNA. Nevertheless, we utilized two control groups to adjust for the confounding effects of smoking on cfRNA expression levels and applied conservative statistical thresholds of 5% FDR and a minimum of 2-fold change difference in expression level between conditions to reduce false positive findings. Furthermore, the fact that we were able to detect cfRNA of hundreds of previously reported RNA transcripts from primary NSCLC biopsies is promising. 

In summary, we have presented transcriptome-wide cfRNA profiling using small volumes of plasma, providing a framework for developing a non-invasive (blood-based) assay for the potential early detection, diagnosis, and monitoring of NSCLC to facilitate high rates of patients able to receive curative surgical resections. Further studies are required for the evaluation of our methodology and its clinical application.

## Figures and Tables

**Table 1:** Demographic and clinical characteristics.

	Cases	Control_ Smokers	Control_ Healthy	*p*-Value Cases vs.		*p*-Value Smokers vs. Healthy
				Control_ Smokers	Control_ Healthy	
**Discovery Cohort (N = 36):**						
Sample Size	12	12	12			
Age (mean, (SD))	67.17 (8.99)	68.44 (10.01)	40.17 (4.99)	0.728	<0.0001	<0.0001
Gender (Male, N (%))	11 (91.67)	9 (75)	7 (58.33)	0.3144	0.0480	0.3144
Race (Caucasian, N (%))	5 (4.67)	5 (4.67)	5 (4.67)	ns	ns	ns
Stage						
Stage I (N)	7 (AC = 5)					
Stage II (N)	4 (AC = 1)					
Stage III-IV (N)	1 (AC = 0)					
Histological Type						
AC (N)	6					
SCC (N)	6					
Average Plasma Volumes Used (mL)	1.6	1.6	1.54	ns	ns	ns
**Validation Cohort (N = 50):**						
Sample Size	25	18	7			
Age (mean, (SD))	64.60 (8.97)	61.28 (10.23)	58.14(17.38)	ns	ns	ns
Gender (Male, N (%))	19 (76.00)	13 (72.22)	5 (71.43)	ns	ns	ns
Race (Caucasian, N (%))	52 (50.99)	11 (61.11)	3 (60.00)	ns	ns	ns
Stage						
Stage I (N)	4 (AC = 2)					
Stage II (N)	2 (AC = 0)					
Stage III-IV (N)	9 (AC = 8)					
Missing Data	10 (AC = 7)					
Histological Type						
AC (N)	13					
SCC (N)	12					
Average Plasma Volumes Used (mL)	0.5	0.5	0.5			

ns—not significant (*p* > 0.05); AC—adenocarcinoma; SCC—squamous cell carcinoma.

**Table 2:** cfRNA differentially expressed in cases compared with both control groups and confirmed by published studies.

Gene Name	Gene ID	Gene Type	Compared with Control_Healthy Group			Compared with Control_Smokers Group			Ref *	%Detected; % CV ^1^	%Detected; % CV ^2^	%Detected; % CV ^3^	Differentially Expressed in Stage I?	Differentially Expressed in Stage II?
			log2FoldChange	*p*-Value	*p*-Adj	log2FoldChange	*p*-Value	*p*-Adj						
ENSG00000102970	CCL17	protein	8.7116	1.5 × 10^−4^	5.2 × 10^−3^	9.1579	5.0 × 10^−5^	1.1 × 10^−2^	[28,29,30]	27.23;	25.00;	41.67;	-	-
		coding								42.76	30.72	56.66		
ENSG00000104880	ARHGEF18	Protein	7.2400	4.6 × 10^−11^	1.4 × 10^−8^	4.2579	4.7 × 10^−5^	1.1 × 10^−2^	[31]	81.82;	91.67;	91.67;	Vs._ control_healthy	
		coding								27.96	20.43	45.07		
ENSG00000123892	RAB38	protein	−5.6801	1.0 × 10^−3^	1.9 × 10^−2^	−6.8853	4.6 × 10^−5^	1.0 × 10^−2^	[32,33]	54.55;	66.67;	41.67;		Vs. both controls
		coding								45.28	48.13	26.48		
ENSG00000178104	PDE4DIP	protein	−5.3701	1.9 × 10^−4^	6.3 × 10^−3^	−5.2635	1.9 × 10^−4^	3.0 × 10^−2^	[34,35]	63.64;	83.33;	50.00;	Vs. control_healthy	
		coding								43.90	36.32	15.50		
ENSG00000259571	BLID	protein	−6.4355	3.0 × 10^−3^	3.7 × 10^−2^	−10.4897	7.4 × 10^−7^	3.1 × 10^−4^	[36]	18.18;	58.33;	33.33;	Vs. control_smokers	
		coding								33.91	53.16	4.89		
ENSG00000271303	SRXN1	protein	−4.8705	3.3 × 10^−3^	3.9 × 10^−2^	−5.9253	2.5 × 10^−4^	3.7 × 10^−2^	[37,38,39]	54.55;	66.67;	41.67;	-	-
		coding								54.83	47.06	27.08		
ENSG00000207586	MIR135A2	miRNA	−19.6805	2.1 × 10^−12^	8.9 × 10^−10^	−25.9452	1.5 × 10^−21^	3.3 × 10^−18^	[40,41]	18.18;	41.67;	8.33;	Vs. both controls	Vs. both controls
										51.99	44.96	40.13		
ENSG00000207639	MIR193B	miRNA	−19.8578	4.3 × 10^−8^	6.2 × 10^−6^	−23.3468	4.0 × 10^−11^	2.1 × 10^−8^	[42,43,44]	18.18;	33.33;	8.33;	Vs. both controls	Vs. both controls
										30.89	52.06	16.35		
ENSG00000207647	MIR153-1	miRNA	−17.9600	1.9 × 10^−7^	2.3 × 10^−5^	−25.6172	1.4 × 10^−14^	1.1 × 10^−11^	[45,46,47,48]	18.18;	33.33;	16.67;	-	-
										26.33	57.10	53.34		
ENSG00000207763	MIR617	miRNA	22.1588	8.9 × 10^−10^	1.8 × 10^−7^	26.8981	2.8 × 10^−14^	2.0 × 10^−11^	[49]	9.09; 33.09	0;0	33.33; 41.96	Vs. both controls	Vs. both controls
ENSG00000207863	MIR125B2	miRNA	12.9574	1.0 × 10^−5^	7.3 × 10^−4^	−10.2390	2.7 × 10^−4^	3.8 × 10^−2^	[50,51,52]	9.09; 31.19	41.67; 51.38	16.67; 32.68	-	-
ENSG00000221552	MIR1303	miRNA	23.9859	3.3 × 10^−11^	1.1 × 10^−8^	39.7024	2.8 × 10^−29^	1.8 × 10^−25^	[31,35]	9.09; 37.13	0;0	33.33; 59.01	-	-
ENSG00000200478	SNORD115-41	snoRNA	−14.1259	1.2 × 10^−5^	8.3 × 10^−4^	−37.1348	3.8 × 10^−33^	1.2 × 10^−28^	[22]	9.09; 14.02	33.33; 43.45	0;0	**	**
ENSG00000212304	SNORD12	snoRNA	−22.5404	4.4 × 10^−10^	1.0 × 10^−7^	−22.3897	2.4 × 10^−10^	1.2 × 10^−7^	[22]	18.18; 68.86	25.00; 40.72	0;0	Vs. both controls	Vs. both controls
ENSG00000255717	SNHG1	processed transcript	−4.2180	1.4 × 10^−3^	2.3 × 10^−2^	−5.2295	5.0 × 10^−5^	1.1 × 10^−2^	[53,54,55]	63.64; 43.01	83.33; 44.07	83.33; 46.29	Vs. control_smokers	Vs. both controls

* References for studies on lung biopsies; *p*-adj—*p*-value adjusted for multiple corrections based on the number of total detected cfRNA transcripts; %Detected—percentage of samples in which the transcripts were detected above threshold; ^1^ control_healthy; ^2^ control_smokers; ^3^ combined cases; ** expressed in opposite direction (upregulated) in control_smokers.

**Table 3:** Enriched pathways in cases compared with the two control groups.

ID	Description of Pathway	Gene Ratio	*p*-Value	*p*-Adjust
**Cases vs. Control_Healthy:**				
GO:0001501	skeletal system development	71/1685	8.5591 × 10^−5^	0.016903
GO:0005125	cytokine activity	42/1656	6.6270 × 10^−5^	0.022704
GO:0005198	structural molecule activity	105/1656	2.0920 × 10^−5^	0.009907
GO:0007186	G protein-coupled receptor signaling	179/1685	5.2099 × 10^−6^	0.002827
GO:0007200	phospholipase C-activating G protein-coupled receptor signaling	22/1685	4.5649 × 10^−5^	0.011269
GO:0007399	nervous system development	258/1685	0.0003	0.037022
GO:0008154	actin polymerization or depolymerization	34/1685	0.0004	0.046481
GO:0009888	tissue development	248/1685	1.0524 × 10^−6^	0.001336
GO:0009953	dorsal/ventral pattern formation	19/1685	0.0003	0.044695
GO:0010454	negative regulation of cell fate commitment	7/1685	2.6616 × 10^−5^	0.007885
GO:0019958	C-X-C chemokine binding	5/1656	2.3133 × 10^−5^	0.009907
GO:0030545	receptor regulator activity	85/1656	2.1418 × 10^−6^	0.002111
GO:0032501	multicellular organismal process	802/1685	2.1589 × 10^−6^	0.002132
GO:0042221	response to chemical	513/1685	0.0001	0.018405
GO:0042246	tissue regeneration	17/1685	0.0002	0.027754
GO:0042692	muscle cell differentiation	55/1685	0.0003	0.037022
GO:0043403	skeletal muscle tissue regeneration	11/1685	0.0003	0.044909
GO:0043503	skeletal muscle fiber adaptation	4/1685	4.4531 × 10^−5^	0.011269
GO:0045165	cell fate commitment	46/1685	1.9621 × 10^−5^	0.006707
GO:0048018	receptor ligand activity	79/1656	2.4643 × 10^−6^	0.002111
GO:0051272	positive regulation of cellular component movement	84/1685	7.8914 × 10^−6^	0.003597
GO:0051493	regulation of cytoskeleton organization	73/1685	0.00012	0.020485
GO:1902903	regulation of supramolecular fiber organization	51/1685	0.0004	0.047617
GO:1904888	cranial skeletal system development	15/1685	0.0003	0.044566
GO:2001046	positive regulation of integrin-mediated signaling	5/1685	6.6284 × 10^−5^	0.014261
**Cases vs. Control_Smokers:**				
GO:0003729	mRNA binding	23/81	1.0069 × 10^−5^	0.001057
GO:0010608	posttranscriptional regulation of gene expression	23/81	3.0028 × 10^−10^	4.69 × 10^−8^
GO:0016441	posttranscriptional gene silencing	22/84	4.4483 × 10^−15^	1.62 × 10^−12^
GO:0016442	RISC complex	23/81	7.5451 × 10^−17^	6.64 × 10^−15^
GO:0016458	gene silencing	23/81	1.5066 × 10^−13^	3.29 × 10^−11^
GO:0031047	gene silencing by RNA	22/84	1.2005 × 10^−14^	3.28 × 10^−12^
GO:0031332	RNAi effector complex	23/81	7.5451 × 10^−17^	6.64 × 10^−15^
GO:0035194	posttranscriptional gene silencing by RNA	23/81	4.3205 × 10^−14^	1.62 × 10^−12^
GO:0035195	gene silencing by miRNA	23/81	3.2204 × 10^−15^	1.62 × 10^−12^
GO:0040029	regulation of gene expression, epigenetic	23/81	7.5803 × 10^−13^	1.38 × 10^−10^
GO:1903231	mRNA binding involved in posttranscriptional gene silencing	23/84	2.5252 × 10^−8^	5.3 × 10^−6^
GO:1990904	ribonucleoprotein complex	23/84	1.9793 × 10^−8^	1.16 × 10^−6^

Gene ratio—number of significant genes identified in the data set as a ratio of the total number of genes in a pathway.

## Data Availability

The data presented in this study are available on request from the corresponding author. The data are not publicly available in accordance with Institutional Review Board-approved protocol guidelines.

## Supplementary Materials

The following supporting information can be downloaded at https://www.mdpi.com/article/10.3390/diagnostics12122897/s1: Table S1—differentially expressed cfRNA; Table S2—genes included in enriched pathways; Figure S1—qRT-PCR analysis of spike-in controls for cfRNA isolation across samples.
