# Supplementary material for: A Pilot Analysis of Circulating cfRNA Transcripts for the Detection of Lung Cancer
Source: Diagnostics (Basel). 2022 Nov 22;12(12):2897. doi: 10.3390/diagnostics12122897 (PMC9776862; doi:10.3390/diagnostics12122897)
Supplement: Supplementary file 1 [file diagnostics-12-02897-s001.zip › Supplementary Figure S1.pdf]

unisp2

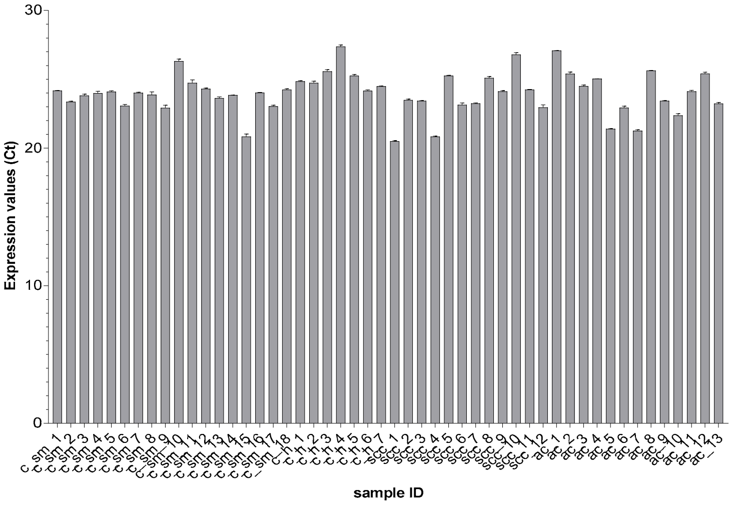

unisp4

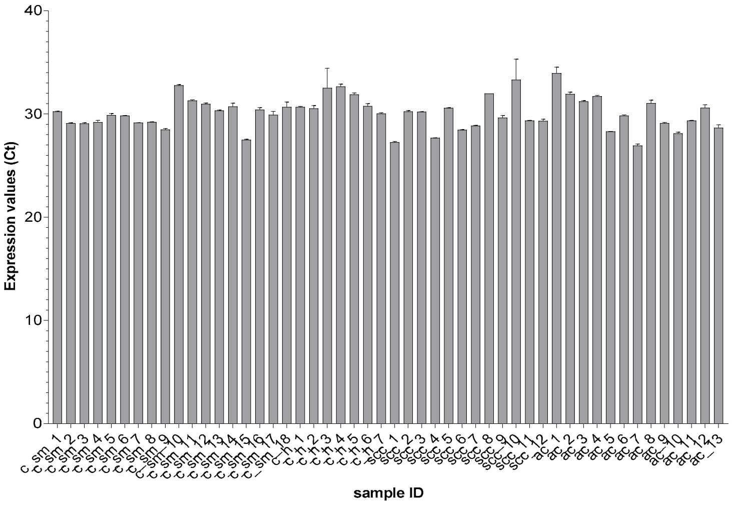

unisp5

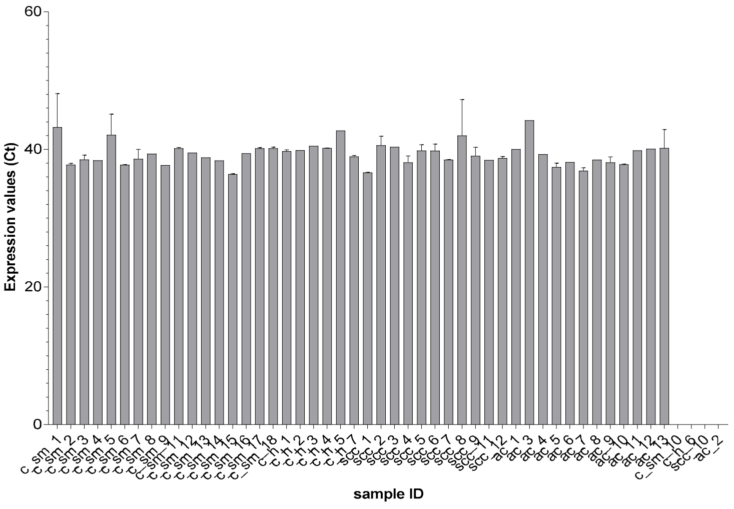

Supplementary Figure S1. qRT-PCR analysis of spike-in controls for cfRNA isolation across samples. Each bar represents mean Ct values averaged for technical repeats. The error bars represent standard error of the mean.
